# The Validation of a Simple, Robust, Stability-Indicating RP-HPLC Method for the Simultaneous Detection of Lamivudine, Tenofovir Disoproxil Fumarate, and Dolutegravir Sodium in Bulk Material and Pharmaceutical Formulations

**DOI:** 10.1155/2022/3510277

**Published:** 2022-02-04

**Authors:** Omobolanle Ayoyinka Omoteso, Marnus Milne, Marique Aucamp

**Affiliations:** ^1^School of Pharmacy, University of the Western Cape, Bellville, Cape Town, 7530, South Africa; ^2^School of Pharmacy, Sefako Makgatho Health Sciences University, Ga-Rankuwa, Pretoria 0208, South Africa

## Abstract

An effective analytical method is requisite to ensure the accurate identification and quantification of drug(s), either in bulk material or in complex matrices, which form part of finished pharmaceutical products. For the purpose of a pharmaceutical formulation study, it became necessary to have a simple, yet robust and reproducible reversed-phase HPLC method for the simultaneous detection and quantification of lamivudine (3TC), tenofovir disoproxil fumarate (TDF), and dolutegravir sodium (DTG) in bulk form, complex polymeric matrices, and during drug release studies. A suitable method was developed using a Kinetex® C_18_, 250 × 4.6 mm column as stationary phase and a mobile phase consisting of 50 : 50 v/v methanol and water with 1 mL orthophosphoric acid, with a flow rate of 1.0 mL/min and column temperature maintained at 35°C. A detection wavelength of 260 nm and an injection volume of 10 *μ*L were used. The method was validated according to the International Conference on Harmonization (ICH) guideline Q2 (R_1_), and the parameters of linearity and range, accuracy, precision, specificity, limit of detection (LOD), limit of quantification (LOQ), robustness, and stability were all determined. Acceptable correlation coefficients for linearity (R_2_) of >0.998 for each of the three drugs were obtained. The LOD was quantified to be 56.31 *μ*g/mL, 40.27 *μ*g/mL, and 7.00 *μ*g/mL for 3TC, TDF, and DTG, respectively, and the LOQ was quantified as 187.69 *μ*g/mL, 134.22 *μ*g/mL, and 22.5 *μ*g/mL for 3TC, TDF, and DTG, respectively. In relation to all the determined validation parameters, this method proves to be suitable for the accurate identification and quantification of the three ARVs, either alone or in combination, as well as when incorporated into polymeric matrices. Furthermore, the method proves to be suitable to detect degradation of the compounds.

## 1. Introduction

**Figure 1:**
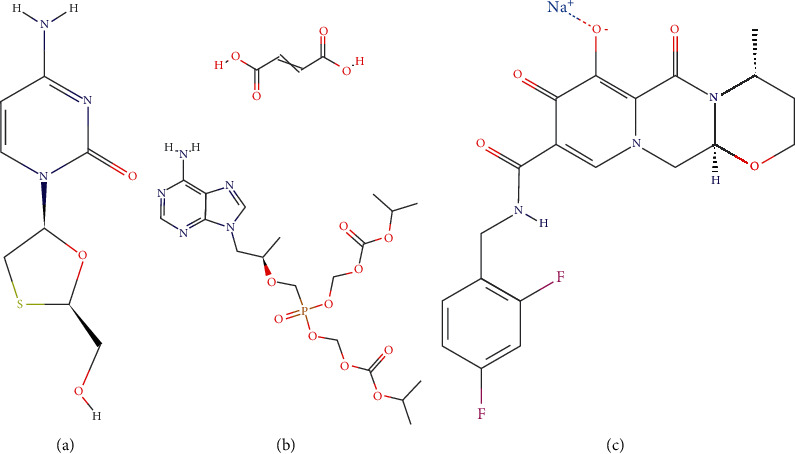
Chemical structure of (a) 3TC, (b) TDF, and (c) DTG [10–12].

Lamivudine (3TC) is a nucleoside analog reverse transcriptase inhibitor (NRTI), used for the treatment of HIV-1, HIV-2, and hepatitis B infection (Figure 1(a)) [1, 2]. Tenofovir disoproxil fumarate (TDF) was the first nucleotide analog reverse transcriptase inhibitor (NtRTI) (Figure 1(b)), approved for the treatment of HIV infection [3]. A combination of TDF with other NRTIs and different classes of antiretroviral drugs (ARVs) causes synergistic effects showing activity against all subtypes of HIV-1 and certain strains of HIV-2 [4]. Dolutegravir (DTG) is a unique second-generation integrase strand transfer inhibitor (INSTI) (Figure 1(c)), developed as a result of the limitations of the first-generation INSTIs, which includes potency, resistance by the virus, dosing frequency, dosing weight, and drug genetic barrier [5–7]. It is effective against numerous HIV-1 and HIV-2 clinical isolates [8]. The 2016 WHO Consolidated Guidelines recommended the combination of the mentioned three ARVs as the first-line regimen mainstay of HIV treatment. Currently, the combination of 3TC, TDF, and DTG is formulated as a fixed-dose combination (FDC) oral tablet.

Although a few FDC formulations, containing these three compounds, have already been developed and marketed, the combination of these ARVs into novel and more patient-orientated dosage forms is steadily emerging. This is mostly attributed to the biopharmaceutical classification system (BCS) classes to which these ARVs belong and the ever-present challenge of reducing the “pill burden” experienced by HIV-positive patients. A previous study has reported the successful development and validation of an RP-HPLC method for the simultaneous detection and quantification of the three mentioned ARVs [9]. Reviewing of this mentioned study revealed chromatographic conditions, which utilised a fairly complex mobile phase gradient, consisting of two mobile phases, as well as the utilisation of two different diluents. However, for the purpose of a pharmaceutical preformulation study, which involved the combination and processing of the three ARVs into complex polymeric matrices, the need was identified to be able to identify and quantify all three compounds simultaneously using a much simpler and more cost-effective method. The rationale for developing this RP-HPLC method was to allow the detection and quantification of 3TC, TDF, and DTG simultaneously across a wide concentration range. Furthermore, the RP-HPLC method should be sufficiently robust and sensitive to detect low drug concentrations in typical drug release media. A thorough literature review did not reveal the availability and reporting of an RP-HPLC method for the simultaneous detection and quantification of 3TC, TDF, and DTG in typical pharmaceutical dissolution media.

In this presented work, the authors describe a simple, yet robust and reproducible RP-HPLC method for the simultaneous detection and quantification of 3TC, TDF, and DTG. This method was validated by proving acceptable limits in terms of concentration range, linearity, precision, accuracy, and specificity of the method towards the accurate identification and quantification of the three ARVs when incorporated into polymeric matrices and typical drug release media as well as the ability to detect unknown degradation products or identify the degradation of the compounds.

## 2. Materials and Methods

### 2.1. Materials and Reagents

Lamivudine (3TC) and tenofovir disoproxil fumarate (TDF) reference standards with certified purities of 99.7% and 99.8%, respectively, were purchased from Industrial Analytical (Johannesburg, South Africa). 3TC bulk raw material was purchased from DB Fine Chemicals (Pty) Ltd (Johannesburg, South Africa), whilst dolutegravir sodium (DTG) certified reference standard (purity of 99.4%), and DTG and TDF bulk raw material was in-kind donations from Cipla South Africa. Chromatography grade methanol (>99.9%) was purchased from Kimix Chemical (Cape Town, South Africa). Ultrapure HPLC water with resistivity of 18.2 MΩ·cm^−1^ was produced by a Lasec® Purite Laboratory Water System (Johannesburg, South Africa). Analytical grade orthophosphoric acid was purchased from Sigma-Aldrich Chemie GmbH (Darmstadt, Germany).

Pharmaceutical grade gelatin and medium molecular weight chitosan were purchased from Sigma-Aldrich Chemie GmbH (Darmstadt, Germany), and pharmaceutical grade xanthan gum was purchased from Savannah Fine Chemicals (Pty) Ltd (Milnerton, South Africa). Distilled water was obtained from a Milli-Q Elix® Essential 3 Water Purification System (Merck, Johannesburg, South Africa). For forced degradation studies, hydrochloric acid (HCl) (32%), sodium hydroxide (NaOH), and 3% v/v hydrogen peroxide (H_2_O_2_), all of the analytical grades were purchased from Sigma-Aldrich (Darmstadt, Germany). All reference and sample solutions were prepared using A-grade volumetric glassware and were filtered into HPLC vials using PVDF 0.22 *μ*m syringe filters.

### 2.2. HPLC Instrumentation and Chromatographic Conditions

The method development was performed using a KNAUER AZURA DAD (Berlin, Germany) HPLC system equipped with an autosampler, quaternary pump, photodiode array detector, and column thermostat. The ClarityChrom software package was used for data processing purposes. A column with end-capped octadecylsilyl silica gel, Kinetex® C_18_, 250 × 4.6 mm (Phenomenex®, Torrance, USA), was used as stationary phase. The chromatographic system was set to run isocratically with a mobile phase consisting of 50 : 50 v/v methanol and water with 1 mL orthophosphoric acid. The flow rate was set at 1.0 ml/min, and an injection volume of 10 *μ*L and detection wavelength of 260 nm were used.

### 2.3. Preparation of Standard Stock and Sample Solutions

A standard stock solution consisting of all three drugs containing 1200.0 *μ*g/ml 3TC, TDF, and 200.0 *μ*g/ml DTG using a 50 : 50 v/v methanol and water mixture was prepared. Several dilutions were made from this stock solution, using the same diluent, and used as working standard solutions for the validation of the analytical method. Sample solutions containing all three ARVs alone and combined with the natural polymers such as gelatin, xanthan gum, and chitosan were prepared in the same concentration range as that of the standard stock solution. These solutions were used in the determination of the specificity and recovery of the analytical method in the instances where the ARVs were incorporated into polymeric matrices.

### 2.4. Method Validation

The method was validated according to the International Conference on Harmonization (ICH) of Technical Requirements for Registration of Pharmaceuticals for Human Use guideline Q2 (R1) and the parameters of linearity and range, accuracy, specificity, limit of detection (LOD), limit of quantification (LOQ), robustness, and stability [13, 14].

#### 2.4.1. Linearity and Range

Linearity and range were measured through the analysis of five serial dilutions of the stock solution ranging between 150.0 and 1200.0 *μ*g/mL for 3TC and TDF and 1.5–210 *μ*g/ml for DTG. For each ARV, the relevant calibration curve was constructed followed by the calculation of the slope, *y*-intercept, and associated correlation coefficient (*R*^*2*^).

#### 2.4.2. Accuracy

The accuracy of this analytical method was determined by preparing a standard solution of a mixture of the three ARVs having a concentration of 600 *μ*g/ml 3TC and TDF and 100 *μ*g/ml DTG. From this solution, three concentration levels of 50%, 100%, and 150% were analysed followed by the quantification of the recovered ARV concentration.

#### 2.4.3. Precision

The precision of this method was determined on two levels, which included repeatability (intra-assay precision) and intermediate precision (ruggedness). Repeatability data for each compound were obtained by analysing six replicates of solutions containing 600 *μ*g/mL 3TC, TDF, and 105 *μ*g/mL DTG. Intermediate precision was determined through the analysis of six replicates of solutions at the same concentration level used during repeatability testing but prepared on varying days and by various analysts.

#### 2.4.4. Limit of Detection (LOD) and Limit of Quantification (LOQ)

The LOD and LOQ concentrations for 3TC, TDF, and DTG when analysed simultaneously were determined following ICH guideline Q2 (R1) by applying the following equations:

(1) $$\begin{array}{c} \text{LOD}=\frac{3.3\sigma }{b},\\ \text{LOQ}=\;\frac{10\sigma }{b}, \end{array}$$

where *σ* is the standard deviation of the response values across the concentration range used to determine linearity and range of the analytical method and *b* is the slope of the calibration curve.

#### 2.4.5. Specificity

Since this method was developed to allow identification and quantification of all three ARVs combined into polymeric matrices followed by drug loading quantification, drug release, and stability testing, it was also considered imperative to validate this method through the combination of the three ARVs with polymers such as gelatin, xanthan gum, and chitosan by adding these polymers to a working solution containing 600 *μ*g/mL 3TC, TDF, and 105 *μ*g/mL DTG. Furthermore, the influence of typical drug release media on the separation and elution of the ARVs was tested by preparing solutions containing all three drugs using pH 1.2 (HCl), pH 4.5 (acetate buffer), pH 6.8 (phosphate buffer), and distilled water as diluents.

Forced degradation studies were performed as per ICH guideline Q1A (R2) stability testing of new drug substances and products [13, 14]. To study possible degradation of 3TC, TDF, and DTG when exposed to an acidic environment, 1 mL of a 1N HCl solution was added to 1 mL of a working solution of an initial concentration of 601.71 *μ*g/ml 3TC, 603.33 *μ*g/ml TDF, and 105.00 *μ*g/ml DTG. This solution was mixed thoroughly and incubated at 60.0 ± 2.0°C for a 30 minute period, followed by the subsequent injection of 10 *μ*L. The same procedure was applied to investigate the possible degradation of 3TC, TDF, and DTG when exposed to alkaline and oxidative conditions, where either 1 mL of a 1N NaOH or 3% H_2_O_2_ solution was added to 1 mL of the stock solution, following the same procedure as described for acidic degradation. In all instances of specificity testing, the obtained chromatography was compared with a standard solution consisting of 600 *μ*g/ml 3TC and TDF and 100 *μ*g/ml DTG.

#### 2.4.6. Robustness

The robustness of this analytical method was performed by doing minor modifications towards the method parameters, which included variation in the column thermostat temperature, different C_18_ column types and manufacturers, mobile phase organic solvent concentration, and detection wavelength.

#### 2.4.7. Solution Stability

The stability of the standard working solution was investigated by storing the solution in the fridge (2°C ± 0.5°C) and in ambient conditions (25°C ± 0.5°C) to determine how stable this solution will remain in the diluent. The standard working solution was stored in the specified temperature conditions for a period of 4 months and was analysed at 0, 1, and 4 months.

#### 2.4.8. Statistical Analysis

All investigated method validation parameters were either performed in triplicate or in sixfold. This allowed the expression of the data as average values with calculated relative standard deviations (%RSD). Regression statistics were calculated using Excel software and applying the Analysis ToolPak.

## 3. Results and Discussion

### 3.1. Linearity and Range

The linearity of the method was established from a regression plot of peak response area against the concentration level of each drug. The linearity was demonstrated across the range of 150.0–1200.0 *μ*g/mL for 3TC and TDF and 1.5–210 *μ*g/ml for DTG, which was evident from the correlation coefficients (*R*^2^) of >0.998 (Table 1), proving that there exists a good correlation between method responses across the concentration range. Further to this, the slope and *y*-intercept were also calculated and are presented in Table 1.

### 3.2. Accuracy

The accuracy of the proposed method was conducted through recovery studies, which were performed by preparing samples at three concentration levels, 50%, 100%, and 150% as outlined in Table 1, falling within the concentration range of 300–900 *μ*g/mL for 3TC and TDF and 53–158 *μ*g/mL for DTG. These solutions were analysed against a reference standard solution of known concentration, and the recovered concentration was quantified and reported.

### 3.3. Precision

On both levels, the analytical method proved to be precise and repeatable with intra-assay precision calculated for 3TC, TDF, and DTG well below %RSD of 2% (Table 1). Intermediate precision, determined across multiple days and by multiple analysts, showed the method to exhibit acceptable intermediate precision with %RSD values less than 2%.

### 3.4. LOD and LOQ

The limit of detection is the lowest concentration of a drug that will be detectable but will not produce repeatable quantification of the specific compound, whilst the limit of quantification is the lowest concentration of the drug that will still be quantifiable with acceptable repeatability. For this method, the LOD and LOQ for 3TC were determined to be 56.31 *μ*g/mL and 187.69 *μ*g/mL, respectively; for TDF, 40.27 *μ*g/mL and 134.22 *μ*g/mL, respectively; and lastly for DTG, 6.77 *μ*g/mL and 22.5 *μ*g/mL, respectively (Table 1).

### 3.5. Specificity

**Figure 2:**
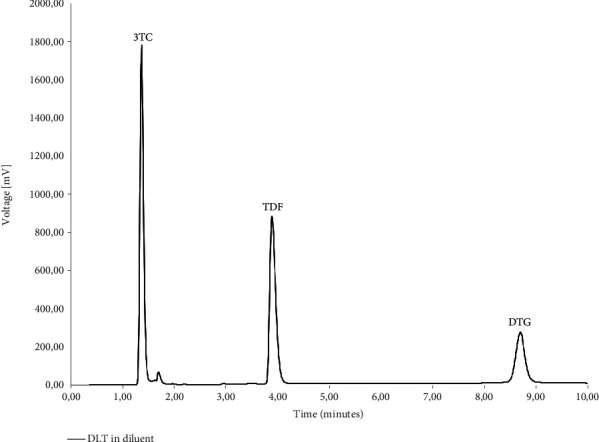
HPLC obtained with 3TC, TDF, and DTG dissolved in the diluent of 50 : 50 v/v methanol:ultrapure water.

**Figure 3:**
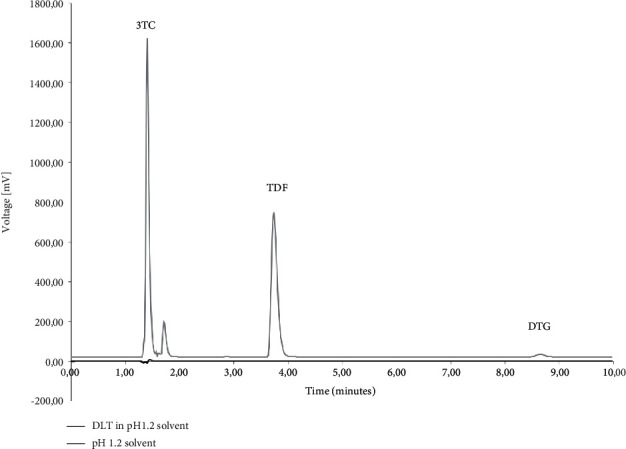
Overlay of HPLC obtained with 3TC, TDF, and DTG (denoted as DLT) dissolved in pH 1.2 buffered aqueous medium and an injection of only pH 1.2 buffered medium.

**Figure 4:**
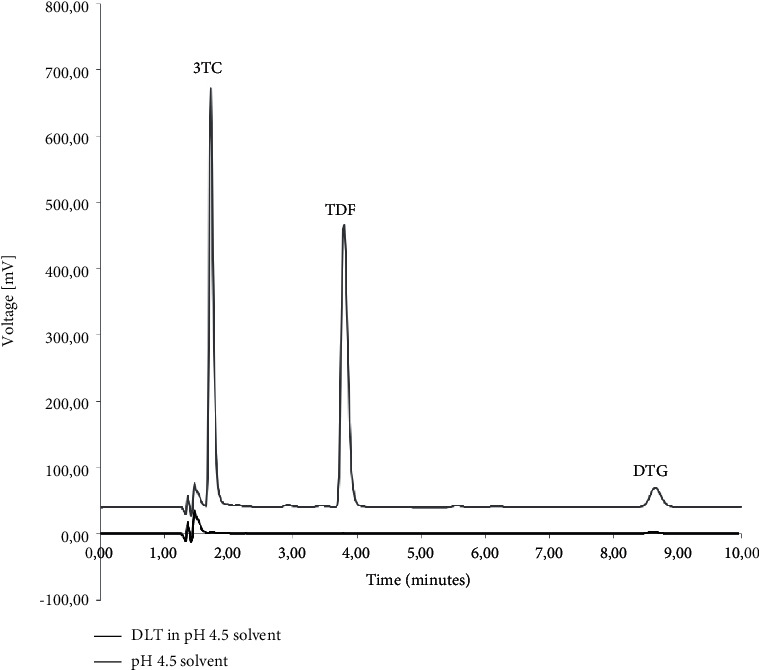
Overlay of HPLC obtained with 3TC, TDF, and DTG (denoted as DLT) dissolved in pH 4.5 buffered aqueous medium and an injection of only pH 4.5 buffered medium.

**Figure 5:**
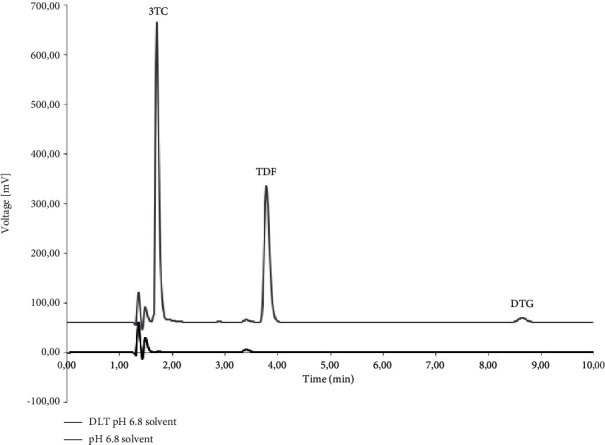
Overlay of HPLC obtained with 3TC, TDF, and DTG (denoted as DLT) dissolved in pH 6.8 buffered aqueous medium and an injection of only pH 6.8 buffered medium.

**Figure 6:**
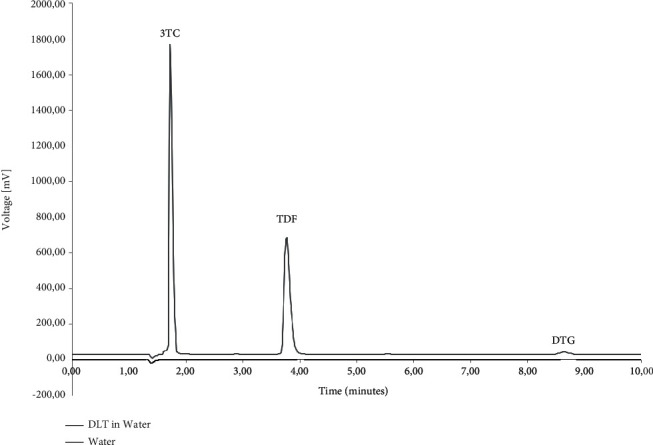
Overlay of HPLC obtained with 3TC, TDF, and DTG (denoted as DLT) dissolved in distilled water and an injection of only distilled water.

The specificity was determined using the diluent, polymers, and typical dissolution media as blank solutions followed by the comparison of these blank injections with the injections of solutions containing the three ARVs.  Figure 2 depicts the chromatography obtained during the simultaneous analysis of 3TC, TDF, and DTG when solubilised in the diluent. During the analysis, it was observed that none of the polymeric materials interfered with the elution of either of the ARVs. The determination of the specificity of the method when buffered aqueous media were used showed no peak interferences when pH 1.2 (Figure 3), pH 4.5 (Figure 4), and pH 6.8 (Figure 5) buffered media and distilled water (Figure 6) are used as solvents. This suggests that this method will be suitable for use as an analytical method during drug release studies in pH 1.2, pH 4.5, and pH 6.8 buffered media and distilled water) of dosage forms containing all three ARVs.

**Figure 7:**
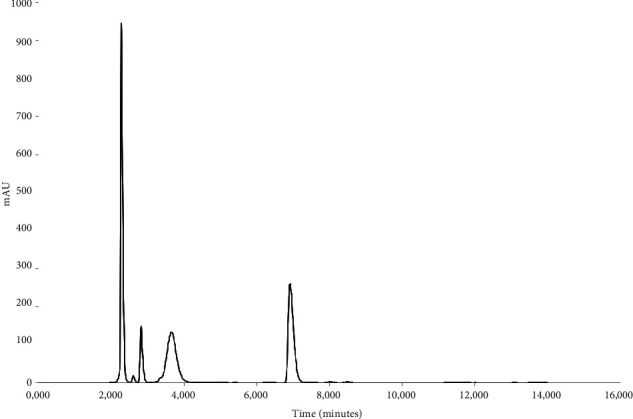
HPLC obtained with 3TC, TDF, and DTG standard solution after treatment with 1N HCl for 30 minutes at 60°C.

**Figure 8:**
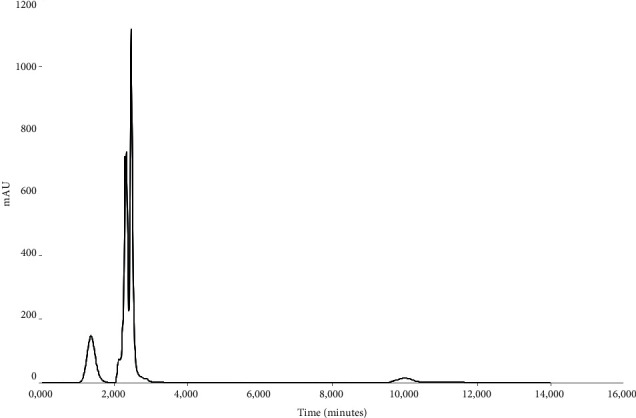
HPLC obtained with 3TC, TDF, and DTG standard solution after treatment with 1N NaOH solution for 30 minutes at 60°C.

**Figure 9:**
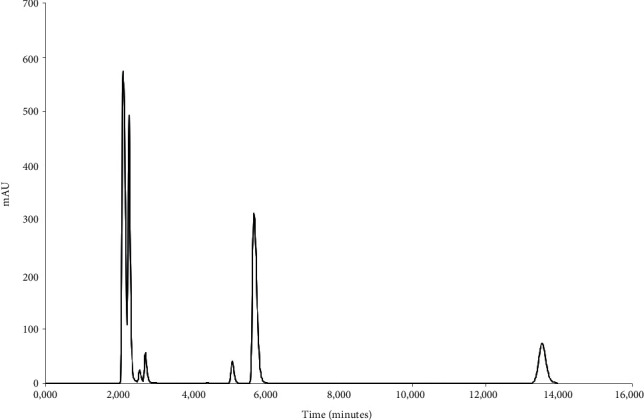
HPLC obtained with 3TC, TDF, and DTG standard solution after treatment with 3%v/v hydrogen peroxide solution for 30 minutes at 60°C.

Forced degradation of the 3TC, TDF, and DTG containing standard solution revealed that this method is suitable and sensitive for the detection of degradation of the three ARVs. After the treatment of the standard solution with 1N HCl, it was observed that 3TC remains quantifiable, but a significant shift in the retention times of TDF and DTG was observed (Figure 7), thus affecting the accurate identification and quantification of these two ARVs. A chromatogram obtained with the standard solution of 3TC, TDF, and DTG is depicted in Figure 8 and shows that none of the ARVs are quantifiable after alkaline hydrolysis. Furthermore, it became evident that of the three drugs, 3TC is sensitive towards oxidation with only TDF and DTG remaining identifiable and quantifiable after treatment of the standard solution with 3% hydrogen peroxide (Figure 9).

Bench top stability of the standard working solution was also conducted, and it indicated the stability of the solution across a four-month period stored at either 2°C ± 0.5°C or 25°C ± 0.5°C (Table 2). This stability study proved that all three ARVs remain stable in the diluent for a period of 4 months when stored at 2°C ± 0.5°C, but when stored at 25°C ± 0.5° for the same period of time only 3TC and DTG remained stable with a 47.05% reduction in the purity of TDF during the storage period. In terms of stability when the solution is exposed to sunlight for a period of 4 months, the potency of both TDF and DTG reduced significantly, as provided in Table 2, thus proving that these ARV containing solutions should preferably be stored at 2°C ± 0.5°C if intended for analysis across long periods of time.

### 3.6. Robustness

The robustness of the method was investigated by applying deliberate changes to the chromatographic system and included changes in the mobile phase flow rate, mobile phase composition, column temperature, and variation between different column lengths. The stability of the analytical solution was also established across a 48-hour period. Throughout robustness testing, a solution at a concentration level of 600 *μ*g/mL 3TC, TDF, and 105 *μ*g/mL DTG was used and an injection volume of 10 *μ*L was used.  Table 3 summarises the results obtained during the robustness testing.

## 4. Conclusion

Based on the precision, linearity, accuracy, recovery, robustness, and specificity results, which includes the investigation into the use of various pharmaceutically relevant solvents and the forced degradation of 3TC, TDF, and DTG obtained using this new RP-HPLC method, showed that this method is suitable for the accurate identification and quantification of the three ARVs. Specificity testing conducted using typical pharmaceutically related drug release media proved that the simultaneous detection and quantification of all three ARVs are not negatively affected when combined with these solvent systems. Since this method is intended for the analysis of 3TC, TDF, and DTG during typical pharmaceutical preformulation and dosage form formulation processes, it was also important to ascertain the suitability of the analytical method when unknown and potential process-induced degradation products form part of the analytical matrix. The specificity and thus suitability of the analytical method to distinguish between 3TC, TDF, DTG, and any unknown degradation products were also proven during method validation. The determination of LOD and LOQ also proved that this method is suitable for the simultaneous detection and quantification of all three ARVs, with significantly low drug concentrations being identifiable and quantifiable.

## Figures and Tables

**Table 1:** Summary of the validation parameters investigated during the validation of the proposed HPLC method for the simultaneous identification and quantification of 3TC, TDF, and DTG.

Validation parameters	3TC	TDF	DTG

Linearity (*R*^2^ > 0.998)	0.999	0.999	0.999
Slope	12236.13	11357.99	29030.74
*y*-intercept	242.59	−100.76	−43.92
LOD (*μ*g/mL)	56.31	40.27	6.77
LOQ (*μ*g/mL)	187.69	134.22	22.5
Accuracy (recovery 98–102%)			
50% level	100.31	101.56	101.27
100% level	100.67	101.33	104.94
150% level	101.57	100.41	100.78
Precision (%RSD <2%)	0.08	0.13	0.17

**Table 2:** Quantification of the percentage (%) purity of each drug after exposure of the working standard solution to 1N HCl, 1N NaOH, and 3% v/v hydrogen peroxide for a period of 30 minutes at 60°C and to storage in 2°C ± 0.5°C, 25°C ± 0.5°C, and normal UV light in ambient conditions for a period of 4 months.

Degradation type	% assay of active ingredient		
	3TC	TDF	DTG

Acid hydrolysis	53.17	36.99	59.21
Alkaline hydrolysis	0.00	0.00	19.92
Oxidation	86.31	38.80	41.26
2°C ± 0.5°C	95.71	101.33	115.31
25°C ± 0.5°C	101.59	52.95	121.01
UV light	101.79	43.22	35.96

**Table 3:** Summary of the effect of deliberate chromatographic variations on the retention time (minutes) and peak symmetry of the peak responses for 3TC, TDF, and DTG.

Chromatographic condition	3TC		TDF		DTG	
	Retention time (min)	Peak symmetry	Retention time (min)	Peak symmetry	Retention time (min)	Peak symmetry
Column temperature (°C)						
35	1.33	1.4	3.35	1.5	7.70	0.8
40	1.35	1.3	3.17	1.4	6.39	1.1
50	1.32	1.4	2.95	1.4	5.36	0.98
Different column types						
Phenomenex^®^ Kinetex^®^ C_18_ 250 × 4.6 mm	1.45	1.4	4.87	1.3	12.08	0.8
Phenomenex^®^ Kinetex^®^ C_18_ 150 × 4.6 mm	1.35	1.3	3.35	1.6	8.20	1.0
Discovery HS C_18_ 150 × 4.6 mm	1.43	1.4	4.85	1.33	12.07	0.8
Mobile phase composition (% v/v)						
90 : 10	1.48	1.40	ND	ND	ND	ND
85 : 15	1.32	3.0	ND	ND	ND	ND
80 : 20	1.51	1.25	ND	ND	ND	ND
70 : 30	1.32	1.38	1.68	1.38	2.30 (0.00)	1.29 (9.61)
60 : 40	1.33	1.25	2.08	1.38	3.63	1.16
50 : 50	1.33	1.4	3.35	1.5	7.70	0.8
40 : 60	1.36	1.5	7.23	2.3	8.42	1.0
Detection wavelength (nm)						
250	1.33	1.25	3.68	1.70	8.60	1.30
260	1.33	1.25	3.68	1.70	8.60	1.30
270	1.33	1.50	3.68	2.00	8.60	1.30
280	1.33	1.50	3.68	2.00	8.60	1.20

ND denotes no peak detected.

## Data Availability

All chromatographic data and methodology used to support the findings of this study are available from the corresponding author upon request.
